# Multifunctionality in Nature: Structure–Function Relationships in Biological Materials

**DOI:** 10.3390/biomimetics8030284

**Published:** 2023-07-02

**Authors:** Jiaming Zhong, Wei Huang, Huamin Zhou

**Affiliations:** State Key Laboratory of Materials Processing and Die and Mould Technology, School of Materials Science and Engineering, Huazhong University of Science and Technology, Wuhan 430074, China; jmzhong@hust.edu.cn (J.Z.); hmzhou@hust.edu.cn (H.Z.)

**Keywords:** biological materials, multifunctionality, multiscale structure, bioinspiration, structure–function relationship

## Abstract

Modern material design aims to achieve multifunctionality through integrating structures in a diverse range, resulting in simple materials with embedded functions. Biological materials and organisms are typical examples of this concept, where complex functionalities are achieved through a limited material base. This review highlights the multiscale structural and functional integration of representative natural organisms and materials, as well as biomimetic examples. The impact, wear, and crush resistance properties exhibited by mantis shrimp and ironclad beetle during predation or resistance offer valuable inspiration for the development of structural materials in the aerospace field. Investigating cyanobacteria that thrive in extreme environments can contribute to developing living materials that can serve in places like Mars. The exploration of shape memory and the self-repairing properties of spider silk and mussels, as well as the investigation of sensing–actuating and sensing–camouflage mechanisms in Banksias, chameleons, and moths, holds significant potential for the optimization of soft robot designs. Furthermore, a deeper understanding of mussel and gecko adhesion mechanisms can have a profound impact on medical fields, including tissue engineering and drug delivery. In conclusion, the integration of structure and function is crucial for driving innovations and breakthroughs in modern engineering materials and their applications. The gaps between current biomimetic designs and natural organisms are also discussed.

## 1. Introduction

**Figure 1:**
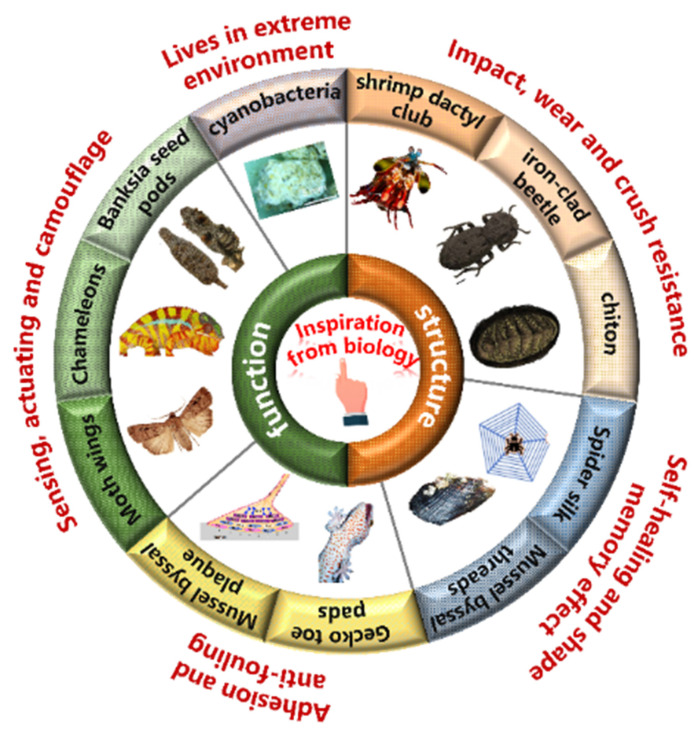
Multifunction in natural organisms. Figures adapted from References [3,11,12,13,14,15,16,17,18,19].

Natural evolution has equipped biological materials in organisms with complex structures and functions that enable them to flourish in harsh environments over hundreds of millions of years [1,2,3,4,5]. Countless generations of adaptation and modification have led to an infinite variety of structures and properties in biological materials, enabling them to perform diverse functions, such as protecting cells and providing structural support for organisms (Figure 1) [1,3,6]. Synthetic materials have gradually replaced biological materials in many applications due to their superior performance in strength, durability, and mature large-scale fabrication strategies. Synthetic compounds have revolutionized several industries, including construction, manufacturing, electronics, and telecommunications, through offering materials with unprecedented properties and capabilities [3,7]. Although synthetic materials have many advantages, they still lack the sophisticated hierarchical structures and multifunctionality presented in biological materials [8,9,10]. Researchers are inspired to explore the unique structures and properties of biological materials and seek ways to incorporate them into synthetic materials to overcome this limitation.

In recent years, researchers have made numerous breakthroughs in the fields of materials science and engineering through the exploration of biological materials. Contemporary advanced characterization and fabrication tools have allowed us to decipher and construct the intricate structures of these materials at different length scales, from the macroscopic to the atomic [20,21]. This has enabled us to better understand the hierarchical structures underlying multiscale biological materials, which consist of a diverse range of building blocks with tightly controlled sizes and shapes. These building blocks include fibrous, gradient, suture, layered, helical, tubular, cellular, overlapping, and more [6,9,21]. The complex design mandates the precise ordering of these structure motifs with varying sizes and shapes into well-controlled arrangements that are reminiscent of walls constructed with individual bricks. The diverse range of building blocks and the coupling between different scales in the hierarchical design of multiscale biological materials are key to their remarkable properties. For instance, the nacre, which possesses a hierarchically ordered multiscale framework composed of rigid mineral tablets and soft organic constituents, exhibits exceptional mechanical properties, such as a fracture toughness value approximately 3000 times higher than the brittle aragonitic CaCO_3_ [22,23,24,25]. Through the study of these multiscale structures, researchers have received insight into optimizing the properties of synthetic materials.

In addition, the multifunctionality of biomaterials has been identified as a pivotal factor in adapting to living environments, thereby advancing the efforts to effectively imitate natural materials and facilitate the development of novel synthetic materials [3,6,26,27,28,29,30,31]. Even though biological materials consist of a limited array of elements compared to synthetic compounds, which are chiefly composed of a few minerals, proteins, and polysaccharides such as chitin and cellulose, the versatility and diversity of properties exhibited by biological materials are remarkable. Recent studies have revealed that the diversity of biological materials stems not only from their composition but also from the complexity of their structures [32,33,34,35]. For instance, collagen is found in connective tissues like bone, skin, and cartilage, providing them with strength and flexibility. It is also present in the cornea to provide structural support and assist in refracting light [36,37,38,39]. Similarly, keratin, a protein utilized by some animals for gripping and snapping tools like nails or beaks, also serves as thermal insulation in wool [40,41,42,43]. Moreover, biological materials exhibit a complex, multi-layered, and multi-scaled structure, where each layer or scale displays unique functionalities [3,6]. This inherent structural complexity enables biological materials to integrate different functions, resulting in exceptional multifunctionality. For example, mussel byssal threads are high-performance fibers which exhibit mechanical properties, while they also possess other functions (such as adhesion and self-healing) [44,45,46,47,48,49]. Similarly, melanin plays a role in coloring skin, hair, and eyes. It also protects against UV radiation while providing mechanical strengthening functions to tissues [50,51,52,53,54]. The understanding of the significant roles that multifunctionality plays in biomaterials has opened new avenues for scientists to create multifunctional materials, which may lead to further innovations and breakthroughs in the design and development of functional materials.

The complex, multi-scale structure of biomaterials has generated considerable interest, but multifunctionality has also emerged as a critical aspect of engineering materials. Therefore, a comprehensive understanding of the diverse functionalities exhibited by biomaterials is essential to optimize and design the versatility and performance of synthetic materials. In this work, we provide an overview of some key functionalities exhibited by biological materials in natural organisms, including impact, wear, and crush resistance; shape memory effects; self-healing properties; sensing; actuation; camouflage; adhesion; antifouling; and living strategies in extreme environments. Different functionalities may hold varying degrees of importance for different applications, and their combination can offer unprecedented advantages and possibilities for biological materials. In summary, this review sheds light on the relationship between the structure and functionality of biological materials, providing a valuable guidance for future research on bioinspired multifunctional materials, which will culminate in the creation of innovative, highly versatile materials.

## 2. Impact, Wear, and Crush Resistance

One of the main functions of biological materials in natural organisms is serving as weapons and defense armors, such as teeth, scales, claws, horns, etc. [55,56,57,58]. To meet the mechanical requirement, the composed materials in biological tissues can be impact, wear, and crush resistant, depending on the loading rate and time scale. For instance, mantis shrimp accelerate their dactyl club up to 20 m/s in less than 3 ms to break the shells of mollusks during their feeding activities, leading to huge impact energy [13,59]. Similarly, bighorn sheep hurl themselves to fight with each other at an impact speed near 9 m/s [60,61]. In contrast, the exoskeletons of insects and crustaceans face quasi-static crush and low-speed impact from the teeth and claws of predators [14,56,62]. In addition, biological tissues such as teeth and bone need to serve for several years, which sometimes cannot be remodeled. This thus requires wear and fatigue resistance in a relatively longer time period. We will take the mantis shrimp dactyl club, iron-clad beetle exoskeleton, and the teeth of chiton as examples to illustrate the structure–function relationship in biological tissues that work at different ranges of mechanical loading rates.

**Figure 2:**
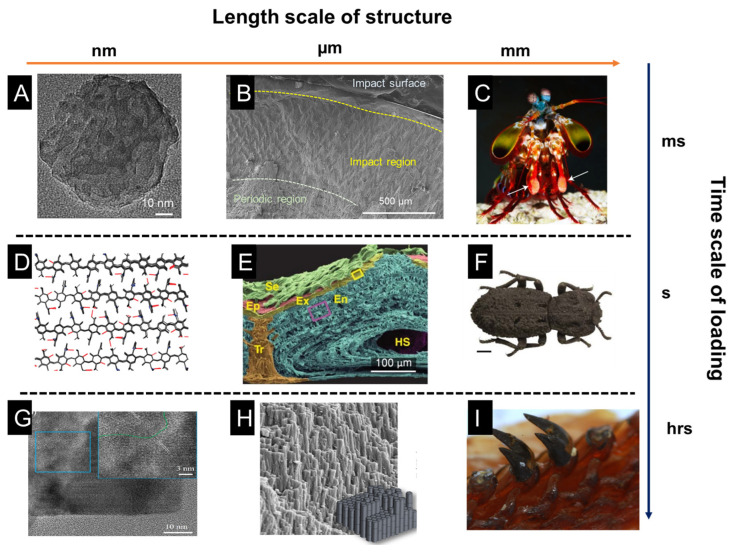
Microstructures of functional tissues in representative natural organisms: the mantis shrimp dactyl club, the exoskeleton of a diabolical ironclad beetle, and chiton teeth. The impact of the mantis shrimp dactyl club last for a few milliseconds, while the crush of the beetle exoskeleton is close to a quasi-static state, and the teeth scratching is a long-time process subject to continuous loading. (**A**) HRTEM of HAP particles after heat treatment at 800 °C. (**B**) SEM micrograph of a transverse section of an intermolted dactyl club. Inset: nanoparticles ~60 nm in diameter are found within the impact surface. (**C**) Photograph of a mantis shrimp and its dactyl club, indicated with white arrows. (**D**) Snapshot of the hydrogen-bonding patterns in α-chitin after 10 ns of equilibration. Hydrogen bonds in blue are to nitrogen and red hydrogen bonds are between oxygen molecules. (**E**) False-colored SEM micrograph of fractured cross-section of the elytra, highlighting leaf-like setae (Se, green), epicuticle (Ep, red), exocuticle (Ex, yellow), endocuticle (En, blue), trabecula (Tr, orange), and hemolymph space (HS, violet). (**F**) Image of diabolical ironclad beetle scale bar. (**G**) HRTEM of the interface between a single-crystal domain and the mesocrystalline core; inset highlights a higher resolution of the boundary between the two domains. It shows a coherent interface between the single-crystalline domains located on the periphery of the mesocrystalline particles. (**H**) Piled rods observed at the tip of a fractured tooth, and an idealized hexagonal rod-like microstructure. (**I**) Radular teeth in chiton. (**A**–**C**) adapted from Reference [13], (**D**) adapted from Reference [63], (**E**,**F**) adapted from Reference [14], (**G**) adapted from Reference [58], (**H**,**I**) adapted from Reference [64].

In Figure 2, a schematic of the length scale of structures and the time scale of loading periods is presented. The impact of a mantis shrimp’s dactyl club occurs within several milliseconds, in which cavitation is observed due to the high-speed impact, leading to extreme damage to hard prey. It has been summarized that the impact resistance of dactyl clubs stems from the gradient and hierarchical structure of dactyl clubs [13]. The inner periodic region is composed of aligned chitin fibers that form a helicoidal pattern, which has been shown to deflect and twist cracks, thus increasing the overall fracture toughness and impact energy dissipation. The outer nanoparticle region is responsible for absorbing the impact energy and preventing penetration. Bicontinuous hydroxyapatite nanoparticles show various energy dissipation mechanisms under high-speed impact: particle translation and breakage, organic fiber bridging, amorphization, etc. Different from mantis shrimp, the diabolical ironclad beetle is well-known for its quasi-static crush resistance [14,63]. It can even survive and withstand the crush of an automobile. Researchers showed the interdigitated suture structure and the laminated chitin fibers provide mechanical interlocking and resist crack propagating through the exoskeleton. In addition to high-speed impact and quasi-static crush, another common loading mode in nature is wear and fatigue, such as in teeth. Chiton teeth are one of the examples showing fabulous wear resistance because of their composition and microstructure [57,64]. Because of its hard teeth, the chiton can scratch off algae from the surface of rocks without severe damage to its teeth. It has been found the nanorod structure at the tip of each tooth provides hardness and fracture toughness. The main composition of the tip of the tooth is magnetite, and the hardness can reach 10 GPa, which is the hardest biomineral found on earth. At the nanoscale, magnetite nanorods are composed of proteins, chitin fibers, and magnetite crystals. The intricate combination of organic and inorganic phases at the nanoscale is formed in the biomineralization process, which is seldom observed in synthetic systems.

In summary, the combination of organic and inorganic phases at different length scales can provide unexpected properties. Structural designs in natural organisms can sometimes overcome the contradictions of mechanical properties, such as hardness and toughness. The mechanical performance at different ranges of loading rates presented by natural organisms looks promising, which stimulates the innovation of high-performance engineering materials.

## 3. Self-Healing and Shape Memory Effect

In the last decade, the scientific community has taken a keen interest in exploring intelligent materials due to their potential to meet the increasing demands of technological advancements. According to the ability to respond to a wide range of stimuli (i.e., heat, light, humidity, pressure, pH, and so on), shape-memory and self-healing materials have been one of the most fascinating intelligent materials [65,66,67,68,69]. In this part, we will summarize the multiscale structure of spider silk and mussel byssal threads and link these structures to the performance of shape memory and self-healing, which will inspire the design and preparation of synthetic materials.

### 3.1. Spider Silk

Spider silk is a remarkable fiber material with impressive stiffness and strength, making it an ideal candidate for various applications [69,70,71,72]. Moreover, it exhibits a peculiar behavior under specific environmental conditions, such as contracting up to 50% of its original length when unrestrained and exposed to water or high humidity (super contraction) [73,74,75,76]. When spider silk is exposed to a stimulus, i.e., water, it undergoes a reversible phase transformation that allows it to recover its original shape. This humidity-sensitive behavior is a typical shape-memory effect.

**Figure 3:**
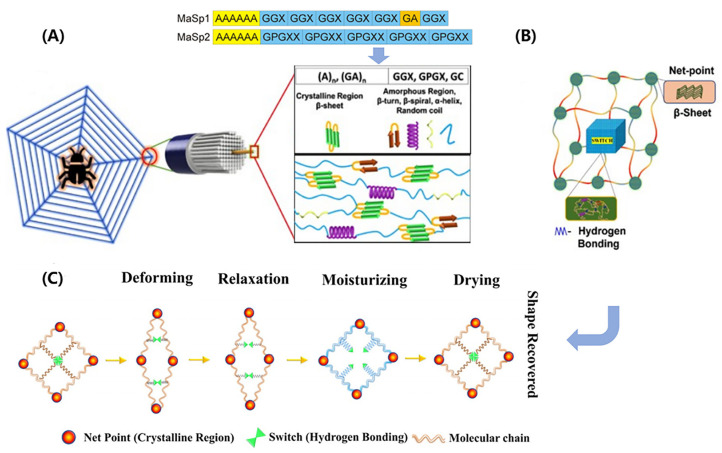
Structure–function relationships in spider silk. (**A**) Schematic of hierarchical and secondary structures. Amino acid motifs: A—Aniline; G—Glycine; P—Proline; X—random amino acid. (**B**) A shape memory structural model. (**C**) Net-point and switch model for moisture-sensitive shape memory mechanism. (**A**,**B**) adapted from Reference [72], (**C**) adapted from Reference [76].

Spider silk is composed of protein molecules arranged in a multiscale structure (shown in Figure 3A), with each level contributing to the final properties [70,71]. In the molecular scale, the proteins are essentially block copolymers, mainly containing two different major ampullate spidroins (MaSp), namely, MaSp1 and MaSp2. They have a large core domain of alternating alanine- and glycine-rich motifs, which are terminated by small non-repetitive amino- and carboxy-terminal domains. The alanine-rich motifs (polyalanine) form stable β-sheet crystallites, while the glycine-rich motifs (GGX, GPGXX) presumably form β-turns, β-spirals, α-helices, and random coils that constitute the amorphous matrix in which the crystallites are embedded [15,73]. At the nanoscale, the MaSp proteins assemble to form intermediate filaments with a diameter of 20–150 nm through simultaneous internal drawdown and material processing. These filaments are further coated with a thin layer (nanometer-scale) of spidroin-like proteins, glycoproteins, and lipids, which protects them from damage and contributes to the mechanical properties of the final material. At the microscale, these coated fibrils are arranged into a final structure with a diameter ranging from 2 to 7 μm.

Spider silk exhibits unique shape-memory behavior, which arises from its synergistic structure comprising highly ordered β-sheet crystals and a less ordered, malleable amorphous network (depicted in Figure 3B). The β-sheet crystals serve as net points, providing the silk with remarkable strength. Conversely, the amorphous network, composed of α-helix, β-turn, and random coil structural elements, confers elasticity upon the silk. Notably, the β-sheet crystals formed by the polyalanine motifs are hydrophobic and less susceptible to humidity, while the elastic amorphous network undergoes supramolecular associations and dissociations of hydrogen bonds, rendering it highly responsive to moisture. Extensive research has been conducted to investigate the contributions of various components in spider silk to its shape-memory behavior [75,76]. It has been found that the amorphous region of MaSp2 plays a vital role in the association and dissociation of hydrogen bonds. This region is rich in prolines, which produce a unidirectional twist and cause steric exclusion effects after external stimulus, disrupting hydrogen bonds in their vicinity and acting as a switch in shape-memory behavior. In particular, a humidity-responsive shape-memory model for spider silk has been proposed, as programmed in Figure 3C [74,75,76]. When the spider silk is stretched, some of the hydrogen bonds break, which deforms the shape of the specimen. The entropy elasticity of the silk causes it to slightly recover. After being exposed to water, water molecules interfere with the hydrogen bonds in the network, causing the silk to plasticize and exhibit super contraction. Upon drying, the hydrogen bonds recombine, causing the silk to recover its original length. This shape memory mechanism has also been observed in other protein-based biological materials, such as keratin.

### 3.2. Mussel Byssal Threads

**Figure 4:**
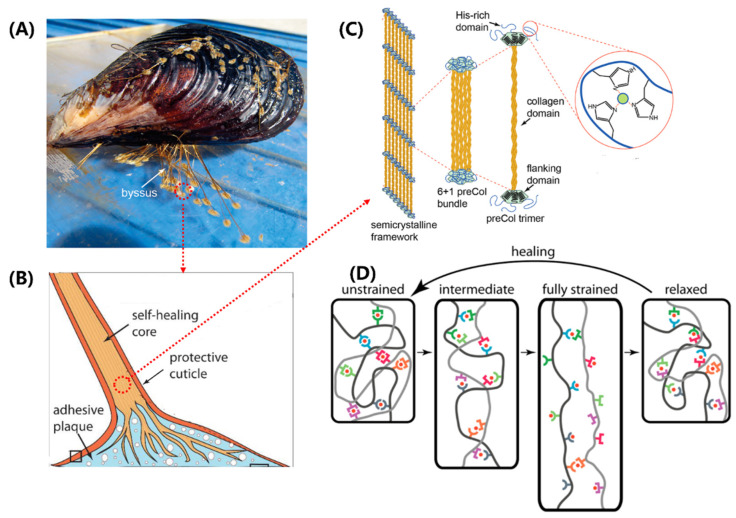
Structure–function relationships in the mussel byssus. (**A**) Mussels attach to surfaces with a byssus. (**B**) The component structure of mussel byssal thread. (**C**) Schematic of the multiscale structure in the core of mussel byssal thread. (**D**) Idealized molecular model of byssal thread deformation and healing. (**A**,**B**) adapted from Reference [45], (**C**,**D**) adapted from Reference [12].

Mussels (Figure 4A) are well-known for their ability to firmly attach themselves to rocky surfaces, nearby shells, and other hard substrates using a proteinaceous attachment device called the byssus [11,45,77,78]. A typical byssus from marine mussels has 50–100 byssal threads composed of numerous self-healing fibers made up of specific protein building blocks. The distal region of the byssal thread is hard, extensible, and tough, capable of dispersing a large amount of mechanical energy through a hysteresis effect. When the mechanical yield is exceeded, the distal region exhibits reduced stiffness and hysteresis effect in subsequent cycles, but after a sufficient rest period, it can fully recover its initial performance. This self-healing behavior is entirely dependent on the specific structure and chemical properties of the protein building blocks that make up the thread [44,46,47,48,49]. The impressive self-healing properties and potential biomimetic applications of the threads of bivalves have attracted considerable attention from researchers, who are studying their molecular architecture and properties to gain insights into how they achieve their remarkable performance.

Mussel byssal threads are composed of three distinct regions, namely the plaque, the core, and the cuticle (Figure 4B), with the core believed to be the primary determinant of overall tensile strength [45]. The core of the thread has a 6 + 1 hexagonal bundle structure consisting of seven triple-helical collagens called PreCols. The central rod-like domain of the PreCol has a typical rigid fibrous collagen [Gly-X-Y]n repeat sequence, approximately 150 nm in length, where X and Y are usually proline or hydroxyproline (Figure 4C). The folded flanking domains of the PreCol are extensible and have different variants, including PreCol-D, an arm-like domain resembling a cable with a polyproline sequence and glycine-rich spacers; PreCol-P, with hydrophobic sequences resembling those of elastin; and PreCol-NG, a highly flexible whip-like domain.

The self-healing ability of byssal threads has been an intriguing topic in biomaterials research. Recent studies have shown that the extensible domains, instead of the collagen domains, are responsible for this property [12,45]. These domains are composed of histidine-rich regions (HRDs) that are capable of binding transition metal ions. The histidine ligands in HRDs donate electrons to form coordination bonds with these ions. Self-healing occurs through the re-formation and exchange of broken metal coordination bonds, leading to the restoration of stiffness and a native-like cross-link network (Figure 4D) [48,49]. Under mechanical stress, sacrificial bond topology offers resistance to deformation and exhibits high stiffness. As the level of strain increases, sacrificial bonds rupture and exchange ligands with neighboring groups, thereby allowing for the extension of length. Eventually, under very high levels of strain, the amino acid ligands are replaced by water molecules. Interestingly, after relaxation, the protein–metal coordination bonds re-form with a less stable topology. They slowly convert back towards the initial structure through the exchange of ligands, which results in more native-like mechanical properties.

## 4. Adhesion and Anti-Fouling

Surface adhesion is a crucial concern in multiple technological fields [79,80,81]. Surface contaminants are one of the primary factors that affect adhesion strength [81,82]. Researchers have been seeking inspiration from nature to solve this issue. Mussels and geckos are two of the most well-known examples of natural adhesion and anti-fouling. Mussels regulate the pH of their surroundings to clean rock surfaces and form robust plaques that adhere firmly to the substrate. They achieve this through employing a variety of molecular interactions, such as hydrogen bonding and metal coordination, to strengthen adhesion [83,84,85,86,87,88]. In contrast, geckos have multi-scale structured toe pads that allow them to develop a sturdy adhesive system. Additionally, geckos’ toe pads display superhydrophobic properties that defend them against contamination [82,83,84,85,86,87,88,89]. Gaining an understanding of the principles that govern natural adhesion and anti-fouling could offer valuable insights for the advancement of new technologies in surface engineering.

### 4.1. Mussel Byssus Plaque

**Figure 5:**
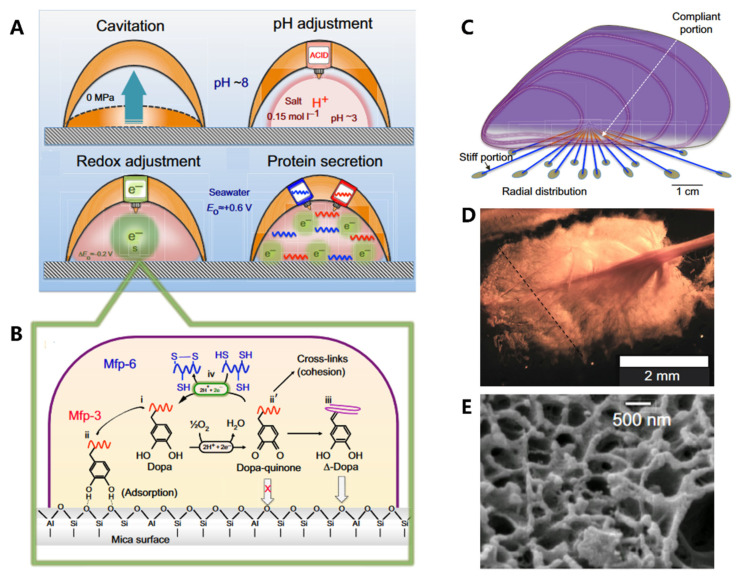
The formation process of plaque and structure–function relationships in the mussel. (**A**) The formation process of cavitation, pH adjustment, redox adjustment, and protein secretion. (**B**) Redox activity is driven by the difference between the high pH and O_2_ concentration of seawater versus the low pH and abundance of electron donors in the plaque. (**C**) Radial distribution of threads. (**D**) The spatulate geometry of a byssal thread and plaque. (**E**) The trabecular (spongy) structure of plaque. Figures adapted from Reference [11].

Marine bivalves, such as clams, mussels, and oysters, are more than a source of food or ornamental shells [11]. They serve as sentinels for the health of coastal ecosystems, providing important ecosystem services that are more relevant in the face of pollution and climate change. Mussels, especially those that form reefs or beds, play an important role as “ecosystem engineers” in coastal environments, similar to the role played by coral reefs in tropical waters. They also help to stabilize sediments, reduce wave energy, and improve water quality through filtering large volumes of water as they feed. Finally, mussels can serve as models for bioinspired technologies, as their unique adaptations to their environment have inspired the development of new materials and designs. For instance, the adhesive properties of mussels byssal threads (Figure 5C) [83,84,85,86,87,88], which they use to attach themselves to surfaces, have been studied for their potential application in surgical adhesives and other biomedical applications.

Adhesion proteins are secreted by the foot’s contact surface, facilitating attachment to various substrates. The adhesion process (Figure 5A) is complex and involves a series of physicochemical interactions between the foot and the substrate. The conditions required for adhesion differ from those found in seawater, such as pH and ionic strength. Organisms can create an environment conducive to the formation of cells and fluids through raising “ceilings” and creating cavitation, where the average pH is approximately 3 and the ion strength is 0.15 mol/L [83]. The pH adjustment process plays a crucial role in cleaning surfaces, killing surface-adhered microorganisms, and regulating the redox environment. The unoxidized form of Dopa is also essential in adhesion, while Dopa-quinone (from oxidation of Dopa) facilitates protein cross-linking [84,85]. Hence, redox adjustment is necessary to control location-specific redox. Despite Dopa-quinone’s excellent cohesive properties, its surface binding characteristics are poor. Therefore, a “self-reduction” process is necessary to reduce Dopa-quinone to catechol (Δ-Dopa), where many of the original dopamine properties can be used again, such as metal coordination, re-oxidation, and hydrogen bonding (Figure 5B) [86]. Finally, the liquid rich in protein solidifies through condensation and becomes plaque.

Researchers have long been fascinated by the mussel’s adhesive properties, which are largely due to the synergistic interplay between the structural components of its byssal threads and adhesive plaques [87,88]. At the centimeter scale, the radial distribution of byssal threads (Figure 5C) in each mussel allows them to withstand dynamic loading, increasing their toughness by up to 900 times. At the millimeter scale, the distinctive spoon-like shape (Figure 5D) of plaques further enhances their adhesion performance. Compared to a cylindrical shape with the same contact area, the spoon-shaped structure improves adhesion by a factor of 20. This is due to the increased contact area and greater surface energy of the spoon-shaped structure, which allows it to create stronger bonds with the substrate. At the microscale, these plaques are composed of a porous solid (Figure 5E) with two distinct length scales of pores, which act to prevent crack propagation, increase energy dissipation, and promote reversible deformation, thereby enhancing the toughness of the adhesive.

### 4.2. Gecko Toe Pads

**Figure 6:**
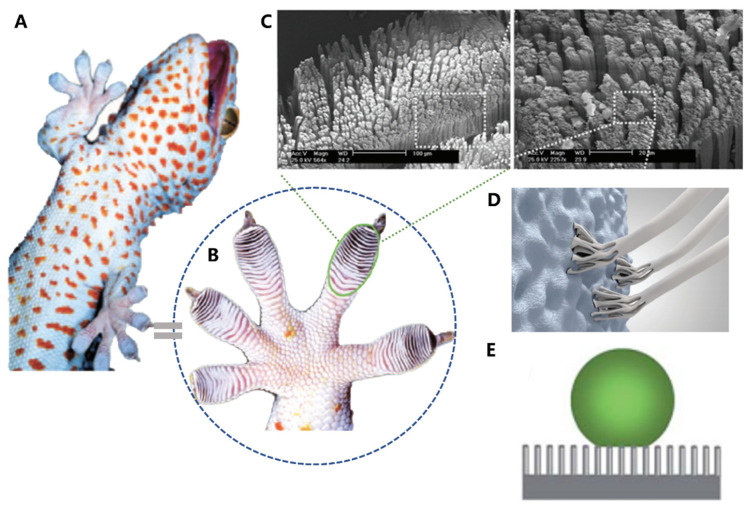
Structure and function in geckos. (**A**) Photo of a tokay gecko. (**B**) Photo of gecko toe pads. (**C**) Pictures showing multiscale structural hierarchy in gecko foot-hair. (**D**) Schematic illustration of structural compliance and adaptation against various rough surfaces. (**E**) Anti-fouling surfaces of gecko toe pads. (**A**–**C**) adapted from Reference [95], (**D**,**E**) adapted from Reference [16].

Geckos (Figure 6A) are fascinating animals found in warm climates, known for their specialized and multifunctional toe pads. These pads possess an efficient reversible adhesive system, which enables geckos to climb almost any surface whether it is rough, smooth, vertical, or inverted [16,89,90,91,92,93,94,95]. In the meantime, the pads also possess superhydrophobic surfaces. This combined performance of high adhesion and anti-fouling has inspired the development of engineering materials such as grabbing robotic hands.

Under microscopic examination, researchers discovered that geckos’ toe pads contain almost half a million keratinaceous hairs or scales [89,90,91,92,93]. Each of these measures between 30–130 μm in length, which is only one-tenth of the diameter of a human hair. These small structures are composed of hundreds of conical protrusions. Additionally, each protrusion ends in a spatula-shaped structure that measures between 200–500 nm in length (Figure 6C) [16].

Recent studies have uncovered the remarkable adhesive ability of gecko toe pads, which is attributed to their hierarchical micro- and nano-structures, which form a periodic array. When the toe pad contacts a surface, the spatula-shaped structures deform, thereby increasing the contact area between the large molecules and converting weak van der Waals interactions into a tremendous attractive force, allowing geckos to effortlessly ascend vertical walls or traverse ceilings [93,94]. The material of gecko foot-hair is composed of relatively hard and hydrophobic β-keratin, including the claws used for mechanical locking (Figure 6D). Geckos have the ability to adhere to surfaces of virtually any roughness and detach with ease at speeds that exceed 1 m/s. In addition to their adhesive properties, the water contact angle of gecko scales (θ) is approximately 160° (Figure 6E), which may be attributed to the micro-roughness of the scales and skin, providing them with self-cleaning properties [16,95].

## 5. Sensing, Actuating, and Camouflage

Given the rapid advancement of intelligent robotics and the increased pressure from modern warfare, the field of research on intelligent sensing and responsive materials has gained significant interest [96,97,98,99]. Consequently, the study of sensing–camouflage and sensing–actuating principles found in biology, such as those observed in moths, chameleons, and Banksia plants, has become a focal point. Moths possess remarkable sensing–camouflage abilities that allow them to blend seamlessly into their environment and evade predators. Similarly, chameleons are well known for their color-changing abilities, which enable them to blend in with their surroundings or communicate with other chameleons [100]. Banksia plants, on the other hand, utilize their sensing–actuating abilities to safeguard their seeds from fire. When exposed to high temperatures and moisture, the plant’s cones open, and the seeds are released.

### 5.1. Moth Wings

**Figure 7:**
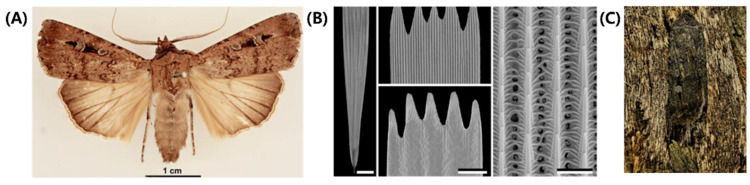
Structure and function in Bogong moths. (**A**) A pinned specimen of a Bogong moth with exposed fore and hind wings. (**B**) Scanning electron micrographs of a forewing scale. Scale bars from left to right: 50 μm, 25 μm and 2.5 μm. (**C**) Bogong moth camouflage on the bark of the Argyle apple. Figures adapted from Reference [17].

Lepidopterans’ wing color is determined by both pigment and structure, which enables them to achieve their camouflage function [17,101,102,103]. In this part, we provide a detailed explanation of how the multi-scale structure of a moth’s wings helps it achieve camouflage. Bogong moths (*Agrotis infusa*) are a species of nocturnal moth notable for their seasonal long-distance migration to the Australian Alps and are shown in Figure 7A [97]. This species undertakes two migrations each year, in the spring and autumn, traveling up to 1000 km to reach their summer and winter habitat. The Bogong moth precisely navigates over such long distances, relying on visual cues and the Earth’s magnetic field for navigation. Research suggests that the moth’s reliance on visual cues and the Earth’s magnetic field for navigation is remarkable. In addition to their unique brownish hue, Bogong moths are also known for their ability to blend in with their surroundings during the day, making them less visible to potential predators in the open plains they traverse.

The wings of Bogong moths are composed of two layers of chitinous scales, as shown in Figure 7B. The well-structured upper lamina is made up of parallel ridges (consisting of slightly overlapping lamellae) and interconnected cross-ribs, leaving minor open windows. Moreover, the top and middle areas of ridges contain a large number of melanin pigments, regarded as brown filters [101,102]. On the other hand, the flat lower lamina is a slightly wrinkled plane that serves as a reflector, with variable thickness. The combination of windows, reflectors, and filters in the scales creates a complex optical system which determines the wing coloration. Additionally, a series of beams and columns act as connection units between the two layers, providing mechanical support and spacing.

When incident light enters the upper layer, most of it passes through the windows. As the film reflector, the lower layer partly reflects the above-transmitted light. Finally, a major fraction of this reflected light reaches again the upper layer, absorbed by a spectral filter (melanin pigments). Hence, the coloration of Bogong moths is not particularly striking, which will match the trees, providing them with perfect camouflage (Figure 7C) [17,103]. Moreover, they will optimize their location and orientation on the bark to maximize their camouflage potential. However, their coloration does not match well with the granite rock in their aestivation caves. As a result, the moths have to tile tightly and form carpets at the cave wall, camouflaged against themselves. In conclusion, Bogong moths have evolved a unique capability of sensing camouflage, which enables them to seamlessly integrate into their environment, making themselves difficult to be detected by predators.

### 5.2. Chameleons

Chameleons are renowned for the ability to change their coloration depending on their surroundings through a complex interplay of pigment and structure color [104,105,106]. Some of them switch colors rapidly in response to outside stimuli (i.e., courtship, male contests, and so on), while others might spend several hours or even days. According to their particular environment, chameleons show distinctive coloration. For example, chameleons living in forested areas obtain more vivid and varied colors. By contrast, chameleons inhabiting arid or grassy surroundings tend to exhibit a brown or tan color. This crypsis, from the ability to sense–camouflage, is their primary defense when facing predators such as birds and snakes [104,107].

**Figure 8:**
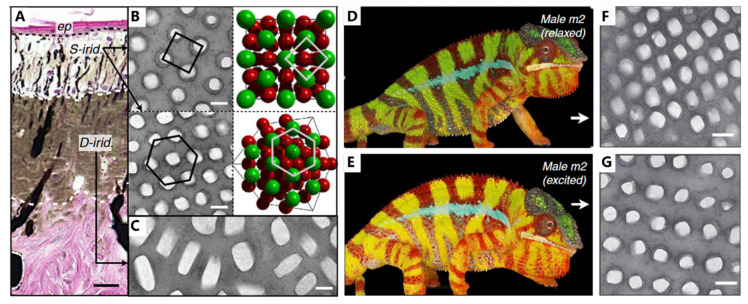
Structure and function in chameleons. (**A**) Hematoxylin and eosin staining of a cross-section of white skin showing the epidermis (ep) and the two thick layers of iridophores. (**B**) TEM images of guanine nanocrystals in S-iridophores in the excited state and three-dimensional model of an FCC lattice. (**C**) TEM image of guanine nanocrystals in D-iridophores. (**D**) The overall color and (**F**) epidermal structure of Male *m2* in a relaxed state (**E**). The overall color and (**G**) epidermal structure of Male *m2* in an excited state. Scale bars, 20 mm (**A**); 200 nm (**B**,**C**,**F**,**G**). Figures adapted from Reference [18].

Chameleons obtain a unique visual system, which permits two eyes to move independently and focus on two different objects simultaneously, forming a full 360-degree arc of vision around their bodies [104,105]. With the aid of visuals, chameleons can perform stereoscopic scans of their surroundings to discover dangerous predators, which is the foundation of camouflage. The mechanism of camouflage in chameleons has been in the spotlight [18,106,107]. It is generally interpreted that these abilities are due to the dispersion/aggregation of pigment. Additionally, recent research has found that the active tuning of guanine nanocrystals and cytoplasm in iridophores is also responsible for the color change. As seen in Figure 8A, the skin of adult male panther chameleons possesses a multilayer structure, with S-iridophores in the upper layer and D-iridophores in the lower layer. S-iridophores (Figure 8B) contain small, densely packed guanine crystals with a higher refractive index, dispersed in the cytoplasm (the lower refractive index materials). This arrangement of high- and low-refractive-index materials acts as a photonic crystal, affecting the color of the skin. Specifically, the distance of guanine nanocrystals is increased in excited male panther chameleons compared with relaxed individuals (Figure 8D,G), presenting a red shift in the reflectance and absorption spectrum, and finally leading to orange skin. On the other hand, D-iridophores with larger, more irregularly shaped guanine crystals, play a crucial role in thermal protection through absorbing and reflecting heat. The combination of S- and D-iridophores plays an important role in thermoregulation, which is helpful for more perfect camouflage. This iridophore system provides a potent example of how structure and function interplay in evolutionary processes and offers implications for the development of intelligent biomimetic materials.

### 5.3. Banksia Seed Pods

**Figure 9:**
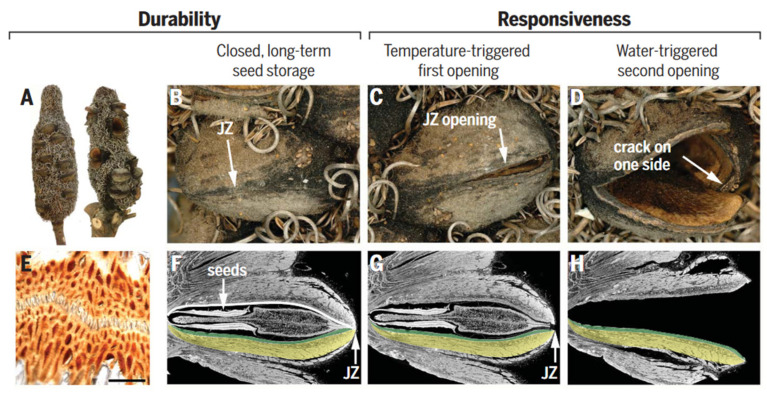
Structure and function of Banksia attenuata. (**A**,**D**) Cones from the North [(**A**), left side] contain mainly closed follicles (**B**). Half-open (**C**) and open follicles (**D**) are frequently found on cones in the South, where opening temperatures are lower [right infructescence in (**A**)]. (**E**) Light micrograph of a junction zone (JZ) sealed with wax (scale bar 100 mm). (**F**,**H**) Virtual cuts through micro-tomographic reconstructions of closed (**F**), half-open (**G**), and open follicles (**H**) showing the seeds with the separator in between [(**F**,**G**)] and the endocarp–mesocarp bilayer (colored in green and yellow in one of the two pericarp valves). The white line in (**F**) indicates the internal valve curvature, which changes with geographic location and climate. Figures adapted from Reference [3].

Many plants in nature have developed strategies to optimize the dispersal and germination of their seeds [3]. Instead of releasing their seeds immediately upon maturity, these plants often wait for optimal conditions for seed dispersal and germination. For instance, the Australian plant genus Banksia stores seeds in metabolically inactive closed pods that can remain viable for up to 15 years (Figure 9A) [108,109,110,111]. Specific environmental stimulations, including temperature and rainfall patterns, trigger the release of these seeds. Different species and individuals may respond differently to these stimulations, depending on their geographic location and climatic conditions. The seed pods resemble small robotic devices, performing a simple task (releasing seeds) after two consecutive signals coming from nature without an external energy source. These processes inspire the design of intelligent soft robots [92,105].

Seed pods are complex structures, composed of a diverse range of materials, including cellulose, hemicellulose, lignin, wax, and tannins, which contribute to both structural and functional roles [108]. These materials determine the pod’s mechanical properties, including strength, elasticity, and resistance to environmental stresses, such as temperature and humidity fluctuations. The pods consist of two pericarp valves, each comprising three distinct layers (i.e., endo-, meso-, and exocarp), with varying orientations of cellulose fibers. These multilayer structures create internal stresses that facilitate seed release. The junction zone, which connects the two valves, is composed of interdigitating cells with a high surface area (Figure 9B,C,E) [3,109]. To prevent water loss and the adhesion of insects or microorganisms, the above structures are coated with wax. During summer days, when temperatures reach about 45 to 50 °C, the wax melts and seals microcracks caused by environmental challenges.

The mechanism of seed release in Banksia attenuata is closely related to the geometric structure of its internal follicles [110,111]. Specifically, the curvature radius of the inner pericarp plays a crucial role in determining the temperature at which seed release begins as well as the size stability of the released seeds. When exposed to high temperatures, the inner pericarp undergoes a softening process, which causes a shift in the internal force balance (Figure 9F,G). This shift triggers the release of stored pre-stress through the formation of cracks and initial openings. Further opening (Figure 9D) of the follicle requires a cycle of wetting and drying to activate the double-layered bending (Figure 9H) of the endocarp and mesocarp.

## 6. Life in Extreme Environments

In previous sections, we discussed how organisms evolve specific structures to realize various functions and adapt to the surrounding environment. There are extreme environments existing on Earth that make life much harder, where prerequisite living strategies are necessary for organisms, such as the deep sea, where there is high pressure and no light; dry desert, where there is very limited water and food; vents of volcanoes, where extreme temperature occurs; etc. [19,112,113]. Interestingly, life has been found inhabiting these extreme environments. It is worthwhile looking into the living strategies of these organisms, which could give us inspiration for inventing new materials or solutions that can help us live in extreme environments, such as Mars.

**Figure 10:**
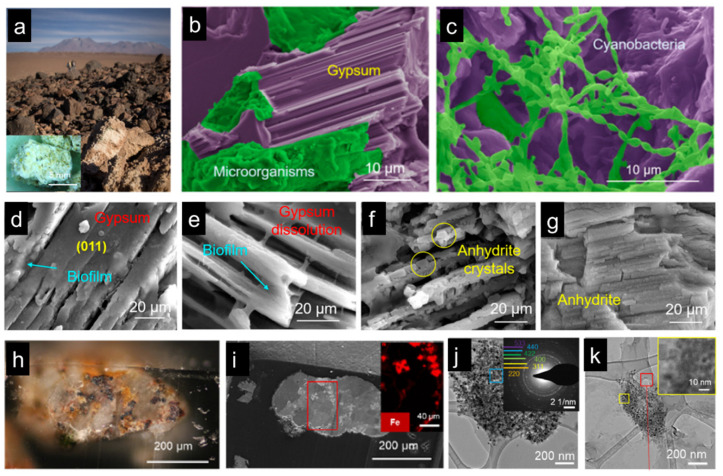
Cyanobacteria living in the Atacama Desert, Chile. (**a**) Gypsum rocks in the Atacama Desert. (**b**,**c**) SEM micrographs show cyanobacteria colonies in the gypsum rocks. (**d**–**g**) The process of gypsum dissolution and phase transformation under the biofilm of the cyanobacteria colony. (**h**,**i**) Magnetite and hematite minerals were found in the gypsum rocks. (**h**–**j**) TEM images show magnetite dissolution and phase transformation. (**a**–**g**) adapted from Reference [19], (**h**–**k**) adapted from Reference [112].

One of the examples is the cyanobacteria that live in the driest non-polar place on earth, the Atacama Desert (Figure 10a–c) [19]. It has been found that due to the lack of water, the cyanobacterium can extract crystalline water from gypsum as its water source and cause the phase transformation of its surrounding rock from gypsum to anhydrite phase. Surrounding the cyanobacteria colony, organic biofilm was detected. The dissolution of gypsum crystals near the organic biofilm was observed, which was believed to occur because of the organic acids existing in the biofilm (Figure 10d–g) [19]. In the meantime, water molecules were released as the dissolution process proceeded. In addition to water, it has also been noticed that cyanobacteria can acquire iron ions from surrounding rocks. Iron is one of the most important trace elements, playing a critical role in the metabolism and photosynthesis process of cyanobacteria. In order to obtain iron, cyanobacteria have to search for iron sources from surrounding minerals, typically magnetite and hematite (Figure 10h,i) [112]. Combining high-resolution microscopy and spectroscopy techniques, it was proved that the biofilms surrounding the cyanobacteria colony could also dissolve magnetite minerals, and iron ions were thus released from the solid minerals (Figure 10j,k) [112]. Additionally, due to the photosynthesis process, oxygen is produced, which turns magnetite to hematite surrounding the cyanobacteria colony. This whole living strategy of cyanobacteria in extreme dry places can provide inspiration for inventing techniques and materials that facilitate human beings’ habitation of space ono planets such as Mars. In addition, advanced techniques such as 3D bioprinting can be used to fabricate functional living materials through utilizing these living organisms as bio-inks.

## 7. Bioinspired Applications and Challenges

### 7.1. Bioinspired Applications

Inspired by the optimized design strategies found in nature and aided by modern nano- and micro- characterization and fabrication technologies, numerous synthetic materials with multiscale structures and functions have been successfully developed [114,115,116,117,118,119,120,121,122,123,124,125,126,127,128,129,130,131,132,133,134,135,136,137,138,139], as shown in Table 1. In this section, we provide examples that mimic the above-mentioned structures and functions.

Cross-scale combined structures comprising soft and hard segments are a common feature found in various biological organisms, such as mantis shrimp, chiton teeth, and ironclad beetles. These structures possess exceptional mechanical properties, enabling them to withstand a wide range of stresses in their natural environments. Drawing inspiration from these stiff–soft composite structures, several methods have been employed to fabricate strong and tough materials [114,115,116,117,118,119,120,121]. Freeze casting provides a route to process materials across multiple length scales. It involves the directional freezing of suspensions containing hard phases, resulting in the formation of a hard scaffold that is subsequently filled with a soft phase, such as PMMA/alumina, PMMA/SiC, and so on [114,115,116]. Another powerful technique for emulating biological structures is additive manufacturing, commonly known as 3D printing [117,118,119,120]. Zhang et al. have successfully produced a porous Al_2_O_3_ scaffold using 3D printing and subsequently immersed it entirely in liquid polyurea to create a hard–soft material inspired by the mantis shrimp [117]. Similarly, Wang et al. have manufactured interdigitated structure plates via multi-material 3D printing technology, mimicking the ironclad beetle [118]. These bio-inspired structures optimize the function of materials, resulting in enhanced mechanical strength, toughness, and increased impact energy absorption.

Appling the dual-phase strategy identified in spider silks and mussel byssal threads, many synthetic, hydro-actuated shape-memory materials have been fabricated [122,123,124,125,126,127,128]. In these cases, the objective was to create net points which are less susceptible to external stimuli and elastic networks with reversible bonding capability. Specifically, Wu et al. have used modified SiO_2_ nanoparticles as net points and formed a double elastic network with reversible dynamic crosslinks and covalent crosslinks to prepare spider-like silk fibers [124]. In this example, reversible dynamic crosslinks are formed through the disruption and reorganization of hydrogen bonds upon exposure to water, while covalent crosslinks are formed after exposure to ultraviolet light, enhancing their dynamic mechanical properties. In addition, modifying and processing biological materials is another effective method for producing bio-inspired shape-memory materials. For example, Kim et al. have reported a spider-inspired silk fiber actuator made of silkworm silk fibroins via microfluidic spinning [126]; Zhang et al. have prepared extensible rTRM7 fibers via an organic solvent-enabled drawing process, recapitulating the self-healing capability in mussel byssal threads [128]. These bio-inspired shape-memory materials can be utilized as smart materials, including applications such as artificial skin, artificial muscle, intelligent sensors, and more.

Inspired by the strategies employed by mussels and geckos, researchers have developed various types of adhesive materials [90,129,130,131,132]. Materials that exhibit satisfactory adhesion under wet conditions, inspired by mussel-inspired dopamine chemistry, have garnered significant attention for medical applications such as drug release and tissue engineering. For example, Hu et al. have proposed a buccal tissue adhesive in the form of a tunable thin film using poly(vinyl alcohol) and dopamine [129]; Wang et al., on the other hand, have developed a dopamine-modified hydrogel wound dressing based on ε-poly-L-lysine-polyethylene glycol, which utilizes horseradish peroxidase cross-linking [130]. Combining inspirations from mussels and geckos, researchers have fabricated several types of dynamic attachment/detachment systems that function underwater. For instance, Lee et al. have reported a hybrid biological adhesive that incorporates gecko-mimetic nano PMMA pillars coated with a dopamine layer, mimicking mussel adhesive properties [131]. This adhesive enables reversible attachment to various surfaces in any environment. In another study, Ma et al. have synthesized an array of mushroom-shaped poly(dimethylsiloxane)/Fe_3_O_4_ pillar arrays decorated with a thermoresponsive copolymer, allowing for controllable movement underwater [132].

Several types of smart skins inspired by chameleons have been developed and fabricated [133,134,135,136,137]. Chou et al. have introduced a stretchable electronic skin that mimics the chameleon’s ability to change color and sense touch [136]. The skin begins with the preparation of an elastic pyramidal-shaped polydimethylsiloxane dielectric layer, which is then spray-coated with single-wall carbon nanotubes to create a pressure sensor (PS). Finally, the PS is combined with an electrochromic layer to create a chameleon-inspired smart skin. Similarly, Kim et al. have developed bio-inspired artificial camouflage skins through integrating a thermochromic liquid crystal layer with vertically stacked and patterned silver nanowire heaters [137]. These skins effectively detect and match the local background color, displaying natural transition characteristics.

### 7.2. Challenges

Synthetic composite materials that mimic the multi-scale structures and multifunctionality observed in nature have made remarkable progress in diverse applications, such as drug delivery systems, wearable electronics, and structural components in the aerospace industry, among others [3]. Specifically, the emergence of advanced manufacturing technologies such as 3D printing has revolutionized the production of intricately detailed structures [1,9]. The capability to fabricate complex geometries and structures has propelled these technologies to the forefront. Through extending the temporal scale dimensions of 3D printing through various approaches, such as utilizing external stimuli like stress fields, electromagnetic fields, and temperature variations, it has become possible to achieve finer structural adjustments and create more impeccable biomimetic designs [9].

Nevertheless, biomimetic engineering materials and structures encounter various challenges [6]. The primary challenge is to achieve precise molecular-scale integration and structural coupling between soft and hard building units at every hierarchical level, while simultaneously balancing mechanical integrity and desired functional properties [26,140]. For example, combining soft and hard materials using multi-material 3D printing presents challenges because the modulus differences between printable materials are often much smaller than those between inorganic and organic phases, thereby hindering the achievement of optimal coupling [5,9]. The second significant challenge involves the rapid and large-scale production of biomimetic materials [141]. For instance, when designing materials at the nanoscale within the 3D printing context, achieving high dimensional accuracy becomes crucial. However, this often renders them incompatible with bulk materials due to the excessively long processing time required [1]. Additionally, biological materials are typically produced and prepared under mild conditions, whereas industrial production often entails multiple steps and specific conditions. Emulating the manufacturing processes found in nature has emerged as a promising direction for future sustainable development [3].

The authors present several recommendations to bridge the gap between biological materials and bio-inspired materials. Firstly, harnessing the power of data science and machine learning can greatly expedite our comprehension of biomaterials, enabling the more precise design of synthetic material structures [142]. This approach has the potential to revolutionize the field through facilitating the exploration and optimization of diverse material compositions, architectures, and properties. Additionally, optimizing 3D printing inks and technologies holds significant promise. Recent advancements, such as the development of organic–inorganic hybrid elastomeric ceramics, demonstrate the potential of this approach [143,144]. Through applying similar strategies to enhance 3D printing inks, we can achieve better integration between soft and rigid structural units, leading to the creation of materials with improved multifunctionality and mechanical properties. Lastly, integrating various micro- and nano-fabrication techniques across multiple length scales is suggested as a viable direction for achieving multiscale structures. Through combining the advantages of different fabrication methods, such as lithography, self-assembly, and additive manufacturing, precise control over features at various scales can be achieved. This integration would unlock unprecedented possibilities in the development of complex materials with tailored functionalities and hierarchical architectures.

## 8. Conclusions

Multifunctional micro- and nano-structured materials play a vital role in diverse fields, including energy, defense, aerospace, and biomedical engineering. Scientists seek to emulate strategies found in nature, where materials exhibit multiscale structures and multifunctional properties that have been refined through evolution. Biological tissues, primarily composed of proteins, polysaccharides, and biominerals, possess a limited variety of chemical components that are biodegradable and recyclable. However, these tissues in natural organisms can optimize specific properties and attain multifunctional integration through employing structural design at varying scales. In the current work, we present several attractive functions realized in natural organisms. However, the contents we discussed here are just the tip of the iceberg; the mysterious functions and marvelous structures of nature are always waiting for us to explore them. Through understanding the mechanisms that underlie biological structures and multifunctionality, researchers can obtain inspiration and develop biomimetic materials that substantially enhance human life while providing valuable insights into designing artificial materials with unparalleled properties and capabilities.

Researchers have employed various advanced techniques, including nanolithography, self-assembly, freeze casting, and additive manufacturing, to fabricate and manipulate materials at different length scales [1,6,9]. Through combining biomimetic design principles with these techniques, they can generate materials that mirror the structure and function of natural materials. One promising method is using synthetic polymers that can self-assemble into diverse shapes and sizes that emulate the hierarchical structures of biological materials. These materials can incorporate functional molecules like enzymes, antibodies, and nanoparticles, enabling specific properties and applications. Another approach is to employ nanoscale building blocks, such as carbon nanotubes, graphene, and metal nanoparticles, to create materials with tailored functionalities and properties. These building blocks can be assembled into intricate architectures like nanocomposites and hierarchical structures to achieve adaptability and multifunctionality.

In conclusion, comprehending structural–functional integration mechanisms in nature can inspire the design and preparation of synthetic compounds with multifunctionality. Optimized multiscale structure and multifunctionality will meet the requirements of the next generation of engineering materials, which tackle the most pressing challenges that our society is now facing.

## Figures and Tables

**Table 1:** Main functions in nature organisms and bioinspired applications.

Main Functions	Representative Organisms	Main Structure and Mechanisms	Bioinspired Applications	References
Impact, crush and wear resistance	Ironclad beetle, Chiton and mantis shrimp	Helicoidal fibers, interdigitated sutures, lamellae	Structural components, cutting tools, armors	[114,115,116,117,118,119,120,121]
Self-healing and shape memory	Spider, mussel	Fibers, reversible bonds, elastic network	Artificial skin, artificial muscle	[122,123,124,125,126,127,128]
Adhesion and anti-fouling	Gecko feet, mussel, lotus leaf	Pillar arrays, dopamine	Medical tapes, robotic arms, coatings	[90,129,130,131,132]
Sensing, actuating, and camouflage	Moths, chameleons, and banksia plants	Hierarchical structure, photonic crystal	Artificial skin, military applications, soft robots	[133,134,135,136,137]
Strategies of living in extreme environment	Cyanobacteria	Biofilms, metabolism, photosynthesis	Living materials, space exploration, and colonization	[138,139]

## Data Availability

No new data were created or analyzed in this study. Data sharing is not applicable to this article.
